# Apoptosis Inducing Factors Involved in the Changes of Flesh Quality in Postmortem Grass Carp (*Ctenopharyngodon idella*) Muscle

**DOI:** 10.3390/foods12010140

**Published:** 2022-12-27

**Authors:** Huaimao Tie, Xuan Lu, Dawei Yu, Fang Yang, Qixing Jiang, Yanshun Xu, Wenshui Xia

**Affiliations:** 1 State Key Laboratory of Food Science and Technology, School of Food Science and Technology, Jiangnan University, Wuxi 214122, China; 2 Collaborative Innovation Center of Food Safety and Quality Control in Jiangsu Province, Jiangnan University, Wuxi 214122, China

**Keywords:** apoptosis, caspases, inducing factors, postmortem quality, grass carp (*Ctenopharyngodon idella*)

## Abstract

Alterations of apoptosis have notable influences on flesh quality, but the mechanism is still unclear. Thus, apoptotic behaviors and related triggering mechanisms need to be explored. Fish muscle was prepared and stored at 4 °C for 0, 24, 48, 72, 96, and 120 h for apoptosis analysis. Results showed that positive apoptotic nuclei were positively correlated with drop loss and negatively correlated with shear force and water holding capacity (*p* < 0.05). Results showed that the triggering apoptotic mechanisms were involved with enhanced transcriptional levels of caspase-2, 3, 7, 8, and 9 through mitochondria and death receptor pathways in the muscle of grass carp. The decreased ATP content, changed cytochrome c redox state, increased protein levels of HSP27 and HSP 90, and enhanced activity of cathepsin (B, L, and D), calpain, and serine proteinase were involved in apoptosis activations. Results indicated that caspases, energy metabolism, cytochrome c redox state, heat shock protein expressions, and protease activities played critical roles in apoptosis alterations in carp muscle during refrigerated storage.

## 1. Introduction

Flesh quality is an important parameter for meeting industrial profitability and consumer acceptability. However, postmortem muscle is accompanied by water loss and texture softening, subsequent aggravated fishy odor, and discoloration, resulting in changes in flesh quality [1]. Thus, investigating the trigger mechanisms and the inducing factors underlying the changes in flesh quality is of great significance. Flesh quality deterioration encompasses a complicated process [2,3], including the undesired chemical, physical, and enzymatic actions and microbial alterations [4]. In the early period of storage, the changes in flesh quality are due to the various enzymatic roles rather than bacterial actions [5]. It was reported that the effects of apoptosis related enzymes could not be ignored [6]. Therefore, it is very important to investigate the activation of apoptosis and the mechanisms in postmortem fish muscle.

Apoptosis often occurs with the loss of membrane asymmetry, cleavage of cytoskeletal proteins, and many other changes, leading to the deterioration of meat quality characteristics [7]. Studies of tilapia muscle have shown changes in apoptosis factors ATP and Bax in postmortem fish muscle [8]. It was reported that apoptosis was associated with the changes in meat quality, including tenderness, water holding capacity, and meat color [7]. Activation of the apoptosis process in skeletal muscles had postmortem effects of meat tenderization and proteolysis [9], and the active caspase-3 occurred along with the degradation of cytoskeletal proteins (troponin-T and desmin) during pork aging [10]. At present, there have been sporadic reports on the phenomenon of apoptosis in fish muscle, such as the activation of caspase-3, 8, and 9 in carp muscle [5], the apoptosis pathway in tilapia [8], and the regulated cytokines gene expressions in postmortem grass carp [5], which need further investigation.

Apoptosis alterations in fish muscle were highly connected with transcriptional regulation by apoptosis related genes, which could be affected by apoptosis stimulates and inducing factors [5]. In the postmortem state, fish muscle cells and tissues are deprived of oxygen and nutrients, resulting in energy deficiency [8]. The changes in ATP had profound changes in mitochondria, including swelling and opening of mitochondrial permeability transition pores (MPTP) [11], which is essential for the cytochrome c release [11]. Recent evidence suggested that apoptosis was highly regulated after cytochrome c release, and the changes in cytochrome c redox state might be involved in this process [12]. Studies also implied that heat shock protein (HSPs) played an important role in regulating cell death [13]. In addition, the mechanisms of apoptosis inducing factors, especially the endogenous enzymes, were involved in yellow cattle [14]. Currently, there is a tremendous lack of knowledge on apoptosis activation and the inducing factors, especially in postmortem fish muscle.

Grass carp (*Ctenopharyngodon idella*), as a great nutritive and economic value fish species in China, has been widely farm-cultured for increasing global production and consumption [15]. Recently, prepared fish under cold conditions was welcomed by fresh on-line shopping platforms and retail supermarkets. Considering the perishable characteristics of freshwater fish muscle even at refrigerated conditions, the shift of its sales model encourages us to better grasp the triggering mechanism of the quality deterioration. Thus, the research on the alterations by the apoptosis process in fish muscle is quite important. Therefore, the object of this research was to explore apoptosis activation in fish muscle for 120 h at 4 °C. Furthermore, present research also investigated apoptosis related molecular mechanisms and apoptotic inducing factors in grass carp, which could provide a mechanism on apoptosis activation and apoptosis inducing factors involved in the deterioration of flesh quality.

## 2. Materials and Methods

### 2.1. Fish Sampling and Storage

For experimental procedures, fish were anaesthetized in a benzocaine 4 g/kg solution before being sacrificed, and then fish death was caused according to a previous study [5]. Dorsal muscle was immediately filleted into 35 g, and then all fillets were stored at 4 °C in individual packages in polyester bags. Fish muscle at 6 h, 12 h, 18 h, 24 h, 48 h, 72 h, 96 h, and 120 h were sampled for analysis. The analysis of fish indicators (gene expressions) were without the storage time of 6 h, 12 h, and 18 h.

### 2.2. Observation of Apoptosis by Hoechst 33258 Staining

Nuclear morphology was analyzed with Hoechst 33258 Staining Kit (Beyotime, Shanghai, China). Fish muscle tissues frozen by liquid nitrogen were cut into 15 μm sections by a freezing microtome (Leica, Nussloch, Germany). The sections were then fixed in 40 g/kg paraformaldehyde at room temperature for 30 min. Subsequently, fish sections were washed with 1 × PBS (pH = 7.4) twice. After drying, the sections were stained by 0.5 mL Hoechst 33258 solution for 5 min and were washed twice by 1 × PBS for 5 min. The stained fish samples were observed in a drop of anti-quenched seal liquid under a fluorescence microscope (Olympus IX71, Tokyo, Japan) at excitation (350 nm) and emission channel (460 nm). The exposure time for each image (0 h to 120 h) was 546 ms, 200 ms, 404.78 ms, 450 ms, 750 ms, and 750 ms, respectively.

### 2.3. Measurement of Shear Force

Shear force of fish muscle was measured using a TA-XT Plus texture analyzer (Stable Micro Systems Ltd., Surrey, UK) according to a previous study [16]. A shearing probe (A/CKB) was cut parallel to the muscle at a trigger force of 5 g, and the compress was set as 50% of the original thickness of the sample. Shear force was the maximum value when samples were cut by the probe.

### 2.4. Detection of Drop Loss

Fish muscle of approximately 3 g was prepared, and the exact weight (W_0_) was recorded. The sample was packed in a vacuum bag and stored at 4 °C for different postmortem times. After storage, the liquid on the surface of the muscle was removed to get the difference of final weights (W_1_). Drop loss was expressed as follows:

(1) $$\mathrm{Drop}\;\mathrm{loss}\;\left(\%\right)=\frac{W_{0}{-W}_{1}}{W_{0}}\times 100$$

### 2.5. Water-Holding Capacity (WHC) Analysis

The methods used for WHC detection were based on those in [17], with some modifications. Fish muscle samples of approximately 3 g (W_0_) were centrifuged at 3000 g for 15 min at 4 °C. The liquid on the fish muscle was drained, and the sample was re-weighed (W_1_). WHC was expressed as follows:

(2) $$\mathrm{WHC}\;\left(\%\right)=\frac{W_{0}{-W}_{1}}{W_{0}}\times 100$$

### 2.6. Gene Expression Analysis

RNA extraction, reverse transcription, and PCR steps were completed on the basis of [5]. Gene-specific primers were prepared by Talen (Wuxi, China) (Table 1) [18]. Amplification of PCR was in a final reaction volume of 20 μL, including Forward Primer (0.4 μL), Reverse Primer (0.4 μL), cDNA (1 μL), RNase-Free water (8.2 μL), and 2X Hieff^®^ qPCR SYBR Green Master Mix (10 μL) (Cat NO.11203, Yeasen, Shanghai, China). The CT value between different storage time groups and the 0 h group were determined. The 2^−ΔΔCT^ method was used to calculate the gene expressions.

### 2.7. Detection of Energy Metabolism Related Products

ATP metabolism related products were detected using HPLC according to the previous study [1]. Energy metabolism related samples were separated by HPLC. On the basis energy metabolism related products of peak area and retention time of the Standards (Sigma-Aldrich Co., Ltd., Shanghai, China), the changes in ATP related products were quantified.

### 2.8. Detection of Cytochrome C Redox State

The methods for cytochrome c redox state were following the procedures of [10], with several modifications. Minced fish sample of approximately 2 g was homogenized in 20 mL cold isolation buffer (50 mmol/L Tris-HCl, pH 7.4). Whole homogenates were centrifuged at 8000× *g* for 15 min. After centrifugation, 250 µL supernatants were used to measure the absorbance by a microplate reader (Epoch 2, BioTek Instruments, Inc., Winooski, VT, USA). Oxidation of cytochrome c was shown as the absorbance at 408 nm, and the changes in cytochrome c redox state were expressed as the absorbance (A408–A550 nm).

### 2.9. Extraction of Protein for Western Blot and Western Blot Procedure

The methods for fish muscle protein extraction and Western blot procedure were carried out with reference to previous research [15]. Total protein of 30 μg was loaded in per lane and separated by 10% polyacrylamide gels, and electrophoresis was performed at 80 V for 32 min, followed by 120 V for 95 min. After electrophoresis, a wet-transfer method was used to transfer the separated target protein to the 0.45 um PVDF membrane (Millipore, Bedford, MA, USA). Blocked membranes were washed three times (10 min) and incubated with primary antibody [HSP27 rabbit polyclonal antibody (Catalog number: 18284-1-AP), HSP90 rabbit polyclonal antibody (Catalog number: 13171-1-AP), beta actin rabbit polyclonal antibody (Catalog number: 20536-1-AP)] at 4 °C for 12 h. Secondary rabbit antibodies (Catalog number: SA00001-2) of HRP-conjugated Affinipure Goat Anti-Rabbit IgG (H+L) were used to incubate membranes for 120 min at room temperature. After incubation, the PVDF membrane was washed three times before visualization. Each experiment was independently carried out for at least three times.

### 2.10. Analysis of Cathepsin B, L, Calpain, and Serine Proteinase Activity

Briefly, homogenized fish muscle 10 multiple (*w*/*v*) were centrifuged at 10,000× *g* at 4 °C for 20 min to collect the supernatant. The enzyme extract of 100 μL was mixed with 90 μL appropriate detection buffer (buffer for cathepsin B and L: 200 mM phosphate buffer, 8 mM EDTA, pH 6.0; buffer for calpains: 20 mM phosphate buffer, 7.5 mM CaCl_2_, pH 6.0; buffer for serine proteinase: 150 mM phosphate buffer, pH 8.0) and 10 μL DTT (6 mmol/L). The enzyme extract, DTT, and detection buffer were completely mixed and were incubated at 37 °C for 10 min. After heating, the mixture was reacted with 100 μL reaction substrate (10 μmol/L). The substrates of Z-Phe-Arg-AMC, Z-Arg-Arg-AMC, N-succinyl-Leu-Pro-AMC, and Boc-Phe-Ser-Arg-AMC from NJPeptide (Nanjing, China) were used as the substrates for cathepsin L, cathepsin B, calpain, and serine proteinase, respectively. The reaction was run at 37 °C for 15 min and stopped with the addition of stopping buffer (3.0 mL), which consisted of 30 mmol/L sodium acetate, 100 mmol/L sodium chloroacetate, and 70 mmol/L glacial acetic acid. The special enzyme-catalyzed release of AMC content was measured using a Cary Eclipse fluorescence spectrophotometer (Agilent, Santa Clara, CA, USA) at an excitation wavelength of 360 nm and emission wavelength of 460 nm, with a slit width of 10 nm. Different concentrations of 100 μL AMC (0 μmol/L to 96 μmol/L) were added with the above detection buffer (100 μL), water (100 μL), and stopping regent (3 mL) to run the specific standard curves, which were mixed and measured by the above fluorescence spectrophotometer. The AMC concentration in fish muscle was calculated by running the specific standard curves. The activities of these enzymes were defined as the release of 1 μmol substrate per min (one unit).

### 2.11. Measurement of Cathepsin D Activities

The changes in Cathepsin D activities were measured using the substrate of hemoglobin. The citrate buffer (0.2 mol/L, pH 3.7) of 1200 μL was thoroughly mixed with enzyme extract (400 μL), and then the mixture was incubated for 10 min at 37 °C. After heating, the enzyme-buffer was reacted with denatured hemoglobin (400 μL, 30 mg/mL) at 37 °C for 60 min. The reaction was stopped with the addition of 2.0 mL stopping buffer of 10% trichloroacetic acid, and then followed by the centrifugation of 10,000× *g* at 4 °C for 10 min to obtain the supernatant for the analysis of protein concentration. Folin-phenol colorimetry was applied to detect the protein concentration of the enzyme extract. The absorbance was measured by an ultraviolet spectrophotometer (UV 1800, Shimadzu Scientific Instruments Inc., Shanghai, China). The activity of Cathepsin D (one unit) was defined as the release of 1 μmol tyrosine per min.

### 2.12. Data Analysis

Software SPSS 19.0 (SPSS Inc., Chicago, IL, USA) was used and one-way analysis of variance (ANOVA) followed by Duncan’s multiple-range test were used to determine significant differences, and *p* < 0.05 indicated a statistically significant difference. The data were presented as mean ± standard deviation (SD) of triplicate determinations.

## 3. Results

### 3.1. Changes in Apoptosis and Flesh Quality

**Figure 1:**
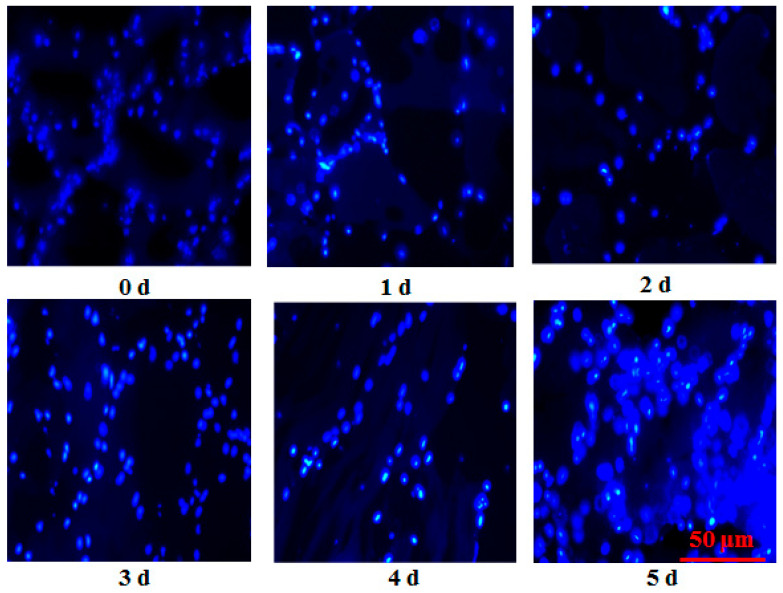
The activation of apoptosis measured by Hoechst 33258 in stored fish muscle under a fluorescence microscopy (×400). Scale bars are 50 μm.

**Figure 2:**
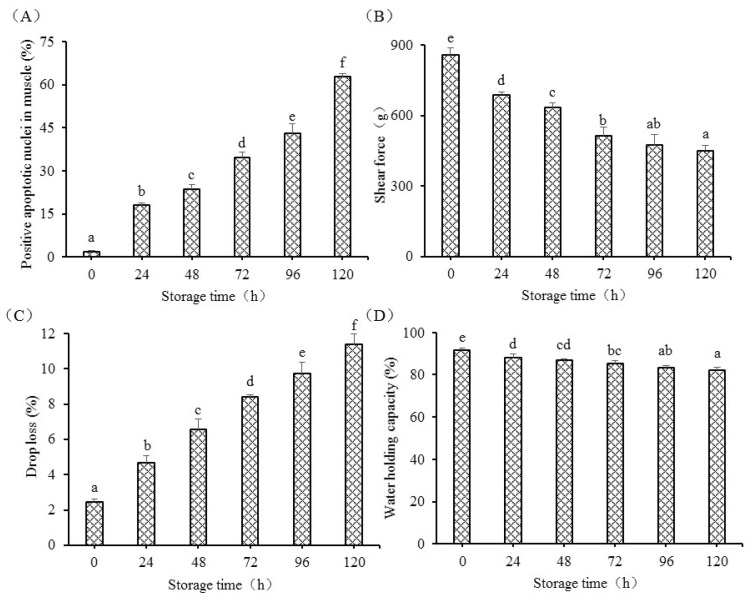
The changes in apoptosis (**A**), shear force (**B**), drop loss (**C**), and water holding capacity (**D**) in fish muscle when stored at 4 °C for 120 h. Results were expressed as the means ± S.D.; Different letters indicated significant difference between different postmortem times (*p* < 0.05).

As illustrated in Figure 1, frozen-section examination showed the activation of apoptosis measured by Hoechst 33258 in postmortem grass carp muscle. At 0 h, the nuclei of normal cells appeared blue. The apoptotic nuclei were almost undetectable from 0 h to 48 h. With postmortem time increasing, positive nuclei in fish muscle cells appeared blue and white with the appearance of fluorescence. The increased white color showed that an enhanced number of apoptotic cells were observed in this study. The morphological characterization of apoptosis is the nuclear condensation and DNA fragmentation. At 96 h, fish muscle cells had an abnormal cell size, irregular shape of cell nucleus, fragmented DNA, and showed the formation of some apoptotic nuclei. The apoptotic nuclei were detected at 72 h during postmortem and were also markedly improved at 120 h. With the extension of storage time, the apoptosis cells numbers showed a gradual increasing trend and reached the maximum level at 120 h during postmortem storage. The changes in apoptosis and flesh quality are illustrated in Figure 2. Time course of apoptosis in postmortem grass carp muscle was markedly increased and obtained the maximum at 120 h. Shear force in postmortem fish muscle markedly decreased from 0 h to 72 h, and also decreased from 72 h to 120 h. The changes in water holding capacity in postmortem fish muscle markedly decreased from 0 h to 120 h (*p* < 0.05). WHC gradually decreased and obtained the minimum at 120 h. As presented in Table 2, positive apoptotic nuclei were positively correlated with drop loss and negatively correlated with shear force and water holding capacity.

### 3.2. Changes in Apoptosis Related mRNA Levels

**Figure 3:**
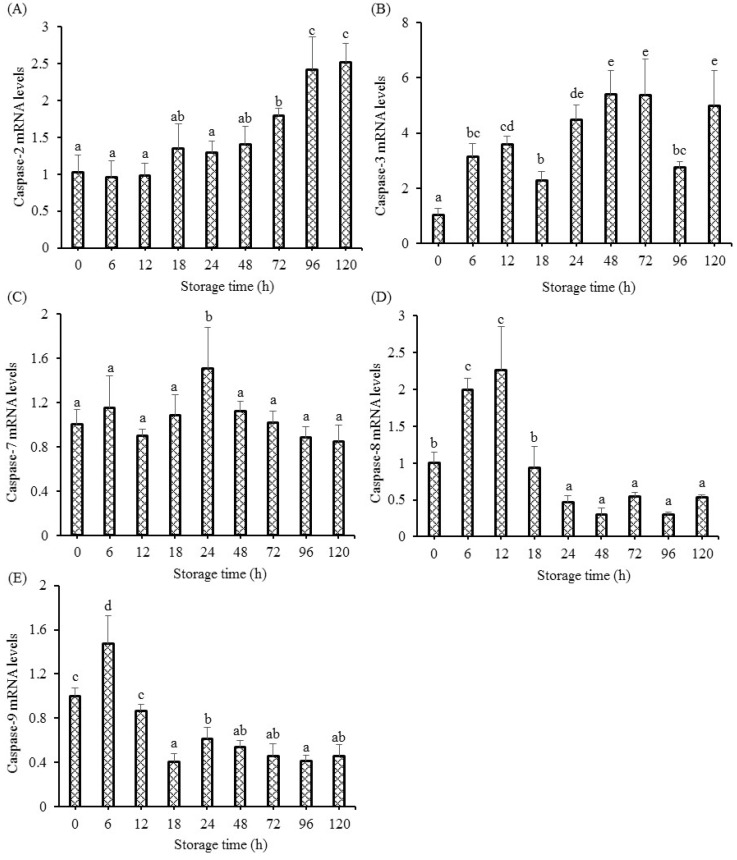
The changes in caspase-2 (**A**), caspase-3 (**B**), caspase-7 (**C**), caspase-8 (**D**), and caspase-9 (**E**) gene expressions in fish muscle when stored at 4 °C for 120 h. Data are shown as the means ± S.D.; Different letters indicated significant difference between different postmortem times (*p* < 0.05).

To visualize the alteration of programmed cell death in postmortem storage, the transcriptional changes in caspases were detected. Postmortem storage affected the gene expressions of apoptotic caspases, which is illustrated in Figure 3. Caspase-2 gene expression insignificantly improved from 0 h to 48 h, markedly improved at 72 h, and showed the highest level at 120 h (Figure 3A). The caspase-3 gene expression notably improved from 6 h to 120 h versus the transcriptional levels at 0 h (Figure 3B). As shown in Figure 3C, caspase-7 mRNA level only notably increased at 24 h, whereas the other postmortem times did not produce significant changes. The transcriptional level of caspase-8 notably increased at 6 h and 12 h (Figure 3D). The gene expression of caspase-8 from 24 h to 120 h significantly decreased. As illustrated in Figure 3E, gene expression of caspase-9 only notably improved at 6 h, followed by a gradual decrease.

### 3.3. Changes in Energy Metabolism and Cytochrome C Redox State

**Figure 4:**
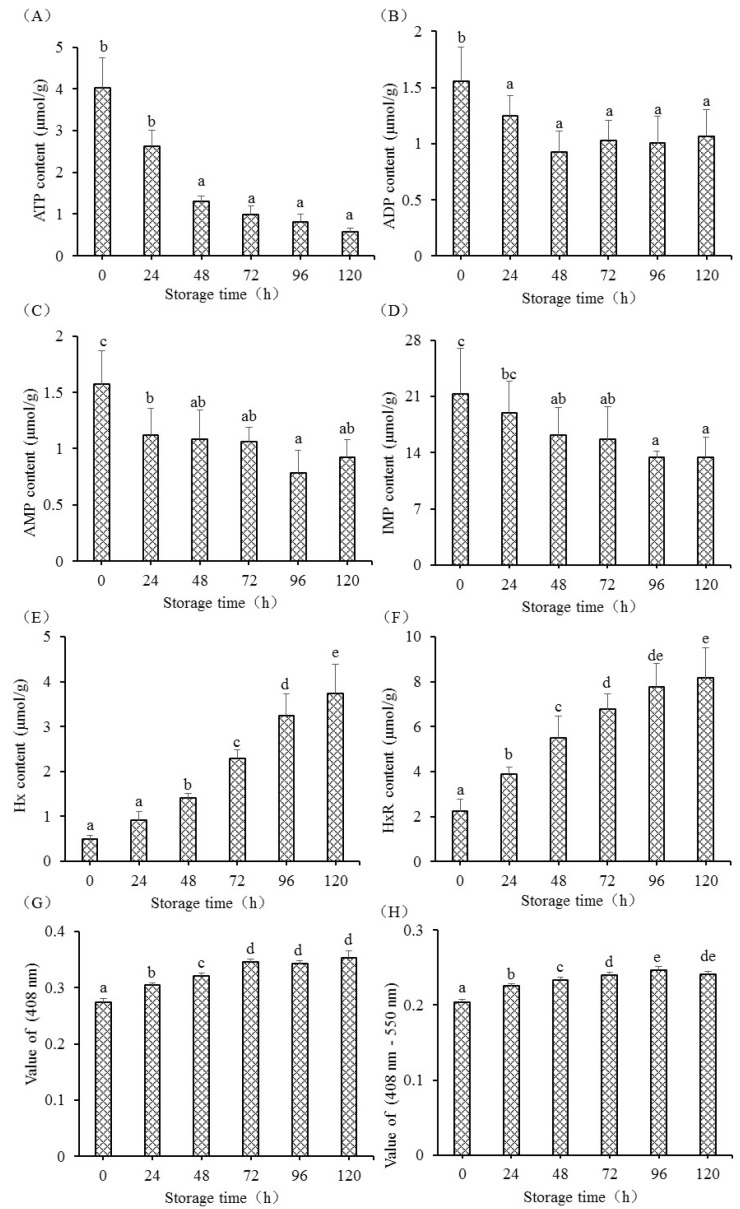
The changes in ATP (**A**), ADP (**B**), AMP (**C**), IMP (**D**), Hx (**E**), and HxR (**F**) contents and the changes in oxidation of cytochrome c (**G**) and cytochrome c redox state (**H**) in fish muscle when stored at 4 °C for 120 h. Data are shown as the means ± S.D.; Different letters indicated significant difference between different postmortem times (*p* < 0.05).

Alteration of ATP related metabolites and cytochrome c redox state are presented in Figure 4. ATP content showed a downward trend and reached the lowest level at 120 h. The content of ADP showed a markedly lower level from 24 h to 120 h (Figure 4B). As shown in Figure 4C, AMP content in fish muscle reduced gradually and reached a minimum at 96 h. IMP content gradually decreased and reached the lowest level at 120 h (Figure 4D). However, the contents of Hx and HxR constantly increased and showed a maximum at 120 h (Figure 4E,F), respectively. The oxidation of cytochrome c notably improved at 24 h and reached a maximum at 72 h (Figure 4G). The cytochrome c redox state showed markedly higher levels as storage time increased (24 h to 120 h) (Figure 4H).

### 3.4. Changes in HSP Protein Expression

**Figure 5:**
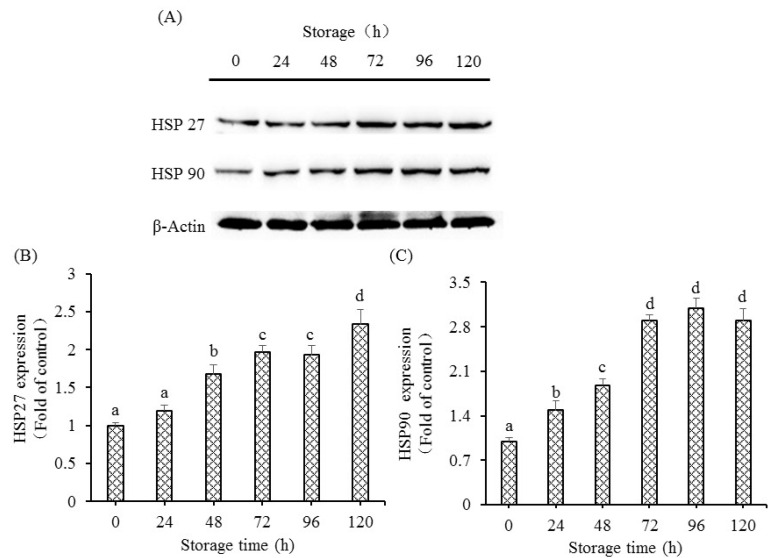
The changes of HSP 27 and HSP 90 protein expressions in grass carp muscle for 120 h storage during postmortem condition. (**A**) Western blotting image of HSP27 and HSP90 in grass carp muscle; (**B**) The expression of HSP27 relative to control; (**C**) The expression of HSP90 relative to control. Data are shown as the means ± S.D. *p* < 0.05 showed significant difference.

The alterations of HSP protein are illustrated in Figure 5. The protein expression of HSP27 notably improved at 48 h and reached the highest expression at 120 h (*p* < 0.05). As postmortem time increased, HSP90 protein levels showed markedly higher levels than that at 0 h, but HSP90 protein levels from 72 h to 120 h did not show a notable difference.

### 3.5. Changes in Endogenous Enzymes

**Figure 6:**
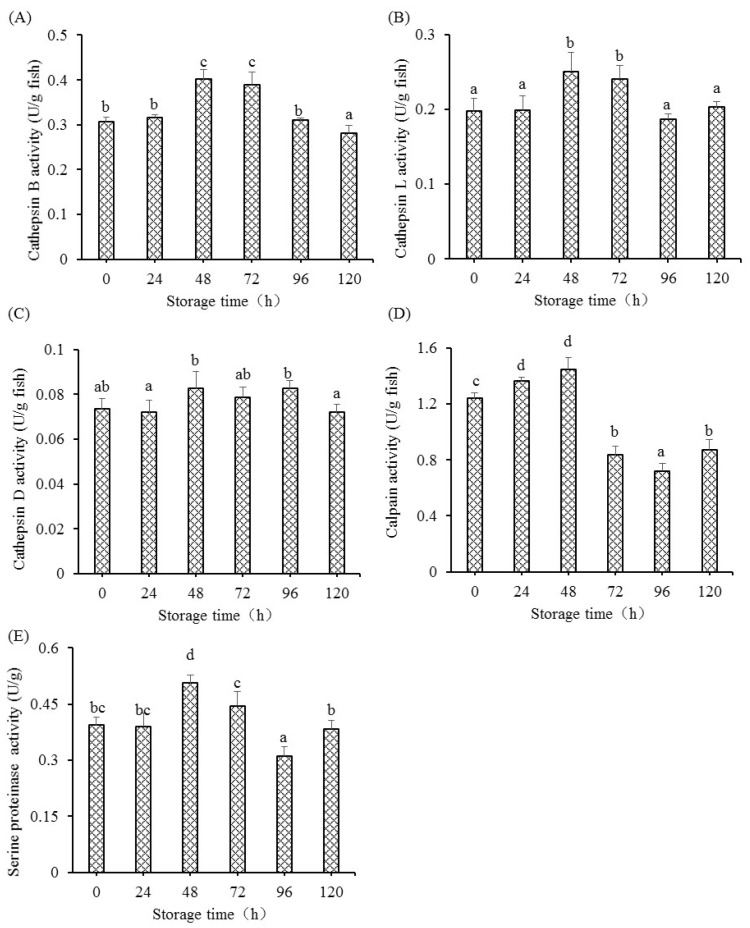
The changes in cathepsin B (**A**), cathepsin L (**B**), cathepsin D (**C**), calpain (**D**), and serine proteinase (**E**) activities in fish muscle when stored at 4 °C for 120 h. Data are shown as the means ± S.D.; Different letters indicated significant difference between different postmortem times (*p* < 0.05).

The changes in endogenous enzyme activity in fish muscle are presented in Figure 6. The activity of cathepsin B markedly increased at 48 h and 72 h, but then declined and had a minimum at 120 h (Figure 6A). The activities of cathepsin L were only significantly enhanced at 48 h and 72 h, but showed no significance from 96 h to 120 h. Cathepsin D activity only significantly improved at 48 h and 96 h, and, for the rest of the storage time, did not show marked differences (*p* > 0.05). Calpain activity was significantly enhanced at 24 h and 48 h (*p* < 0.05), but did not show a significant decrease at 72 h (*p* < 0.05). Serine proteinase activity only markedly improved at 48 h, but then showed a gradual decrease and showed the lowest activity at 96 h.

As presented in Table 3, positive correlation analysis between HSP27, HSP90, caspase-2 and oxidation of cytochrome c and positive apoptotic nuclei were observed in postmortem fish muscle (*p* < 0.05).

## 4. Discussion

Apoptosis was detected in postmortem fish muscle using Hoechst 33258 from 0 h to 120 h. Chromatin condensation is the hallmark of apoptosis. The enhanced blue to white color also showed an increased activation of the apoptosis process in postmortem fish muscle from another insight, which was supported by the previous study evaluated by HE observation [5]. This study observed the irregular shape of cell nuclei, fragmented DNA, and the formation of some apoptotic nuclei, indicating that apoptosis alterations were involved in postmortem storage. Green showed that the apoptosis process usually ranges from several minutes to several days [19]. The apoptotic nuclei markedly increased from 0 h to 120 h and showed that the apoptosis process in grass carp muscle takes several days. The apoptotic nuclei were positively correlated with drop loss and negatively correlated with WHC and shear force, indicating that apoptosis was tightly connected with the changes in flesh quality. The present study was supported by a study in duck, which showed that increased apoptosis was associated with the changes in meat quality, including tenderness, water holding capacity, and meat color [7]. The changes in shear force in fish muscle could be explained by the role of caspase-3. Apoptotic inhibitor could reduce mitochondrial apoptosis activation and decrease structure protein degradation in postmortem muscle [20].

The present results also demonstrated that apoptosis initiation occurred in postmortem fish muscle, which was consistent with the results observed in bovine longissimus muscle [21] and in grass carp [5]. However, our previous study observed a decrease in caspase-3 activity in fish muscle [5], but the present study observed increased mRNA levels of caspase-3, implying the transcriptional regulation failed to regulate caspase-3 at the later postmortem storage. The results in bovine showed that the increase in apoptotic cells was observed with storage time ranging from 6 h to 168 h, and the postmortem tenderization was closely associated with markedly enhanced activation of apoptosis after 24. However, these results were different from those in poultry muscles, where apoptosis reached its ultimate point at approximately 8 to 12 h postmortem in duck muscle [7]. The reason is likely due to the species of animal and different fiber types.

A previous study in grass carp muscle showed increased DNA fragmentation in fish muscle [5], which was supported by this study. The fragmented DNA is regulated by the specific endonuclease of DNA caspase-activated DNase (CAD), which is explicitly activated by caspase-3 [22]. The activation of CAD causes the breaks with 3′-hydroxyl ends by generating a double strand and results in the condensation of chromatin and the degradation of chromosomal DNA [23]. Apoptosis, as a relatively precise program, is characterized by the phase of initiation and the execution by effector caspases. The activation of initiation caspases successively activates the effector caspases, but this process is also dependent on the type of muscle cell and the nature of the stimulus. The activation of caspase-2, 3, 7, 8, and 9 could initiate apoptosis in mammals, which leads to the cell disruption phase, including the formation of apoptotic bodies and cytoskeletal reorganization [24]. This study observed up-regulated caspase-2, 3, 7, 8, and 9 gene expressions in fish muscle, implying that fish cell apoptosis was aggravated by caspases during the postmortem storage. The changes in gene expressions of caspase-8 and 9 showed the same changing trend as our previous study [5]. The initiation of caspases and the apoptosis procedure needs sufficient intracellular ATP. Under conditions of sufficient intracellular ATP, the way of death for cells is through apoptosis rather than necrosis [25]. There is a decrease in energy metabolism in postmortem fish muscle, leading to a rapid decrease in ATP at 24 h, which was synthesized by ADP and creatine phosphate. This research observed the initiation of apoptosis and the up-regulation of caspase mRNA levels in grass carp muscle, indicating that the oxygen deprivation induced intracellular ATP was sufficient at least for initiating the apoptosis procedure especially at the early stage, which was in accordance with the results in pork muscle [10].

Oxygen deprivation induces the mitochondrial pathway and the death receptor pathway [26]. The mitochondrial pathway is characterized by the regulation of apoptosis related factors, including pro-apoptotic members (Apaf-1 and Bax), anti-apoptotic members (Bcl-2, Mcl-1, and IAP), and caspase-9 activation. Activated caspase-9 causes the activation of the executioner caspase (caspase-3 and caspase-7) reactions [27]. The up-regulated gene expression of caspase-9 was observed in postmortem fish muscle, indicating that the initiation of apoptosis cascade reactions in muscle was involved with the mitochondrial apoptotic pathway. The death receptor pathway transmits external signals, cleaves caspase-8, and activates caspase-3 in human erythrocytes [28]. The transcriptional levels of caspase-8 and executioner caspases (caspase-3 and caspase-7) were enhanced in grass carp muscle, suggesting that the activation of apoptosis cascade reactions was involved with the death receptor pathway. Caspase-2 was involved in apoptosis affected by heat shock. On the condition of stress-induced apoptosis, including heat shock, cytoskeletal disruption or DNA damage could lead to the recruitment of caspase-2 [29]. The enhanced protein levels of heat shock in this study might have contributed to the increased gene expression of caspase-2 at 72 h to 120 h. The caspase-8 and caspase-9 initiations were observed at 0.25 d, indicating that apoptosis initiation (death receptor pathway and mitochondrial pathway) occurs at the early postmortem process. Once the effector caspases (caspase-3 and caspase-7) are activated, the execution of apoptosis reactions is started, which is characterized by cleaving specific substrates. Significantly enhanced mRNA levels of caspase-3 (6 h to 120 h) and caspase-7 (24 h) were observed in fish muscle, indicating that caspase-3 might be the main executioner in fish muscle.

The production of ATP in the metabolic pathway is supplied by mitochondria. Study in tilapia muscle during storage observed the rapid decrease in ATP, indicating a decrease in energy metabolism [8]. A study also showed that mitochondria underwent profound structural and dysfunctional changes during storage of oxygen and energy deficiency [30]. Furthermore, mitochondrial impairment had the ability to trigger the initiation of apoptosis by the apoptosis pathway [17,31], which was due to the opening of the mitochondrial outer membrane and the subsequent release of mitochondrial inter-membrane localized proteins [26]. With the opening of the mitochondrial permeability transition pore (MPTP), mitochondrial localized protein, including cytochrome c, released to cytoplasm to activate apoptosis and eventually cause cell death by the mitochondrial apoptotic pathway [32,33]. However, the apoptosis process was highly regulated even after cytochrome c release, which was initiated with the changes in the cytochrome redox state [12]. Thus, the changes in the cytochrome redox state is important for the apoptosis process. In apoptotic cells, the released cytochrome c in cytosol was rapidly oxidized by cytochrome oxidase [12]. Research indicated that reduction of cytochrome c had no capacity to induce the alteration of apoptotic protease, whereas oxidization of cytochrome c had the ability to cause the rapid activation of caspases [34]. This research showed an increase in oxidation of cytochrome c, indicating that cytochrome c was in the direction of oxidation. Results of cytochrome c redox state also showed the markedly higher level of cytochrome c during postmortem storage, indicating that the relative changes in cytochrome c redox state (oxidation—reduction) and the subsequently activation of the apoptosis process in postmortem muscle (24 h to 120 h), which was supported by the previous study, showing that the oxidation level of cytochrome c increased during aging and thus mediated apoptosis [10]. Oxidized forms of cytochrome could combine with apoptotic activating factor-1 (Apaf-1) to cause the formation of a complex of apoptosome, which finally resulted in the activation of caspase-9 [12]. Moreover, the activated caspase-9 could activate the downstream executioners (caspase-3) [34]. Therefore, the changed cytochrome c redox state in fish muscle should be responsible for the increased apoptosis.

When cells were in danger, the chaperone proteins of heat shock proteins (HSPs) appeared as soon as possible. Based on the molecular weight, the HSPs are divided into numerous subfamilies. On the condition of stressors, proteins of HSPs help in the maintenance of the internal environmental integrity of cells and protection of cell survival [13]. Stress proteins have an anti-apoptotic role in programmed cellular death, and stress could induce and affect anti-apoptotic roles of HSPs [35]. However, under conditions of intense stress, HSPs have the ability to generate cell death via the mitochondrial pathway to active caspases (initiators or effectors) [36]. The present study observed the enhanced protein expressions of HSP27 and HSP90 in fish muscle, showing that HSPs respond to either the activation of apoptosis process or the stress itself. However, the stress after slaughter is very intense. Based on the above results, HSPs might exert the role of inducing cellular death rather than protecting target proteins from degradation by effector enzymes, and the markedly enhanced HSP27 and HSP90 protein expressions might be one of the important apoptosis inducing factors during postmortem alterations. The increase in HSP in postmortem fish muscle was supported by the previous study, showing that proteins of some HSP could prevent the formation of oxidation and have a positive relationship with postmortem tenderness. The activation of high molecular weight of HSPs was ATP-dependent chaperones, whereas the activation of low molecular weight of HSPs was ATP-independent chaperones [36]. Our results showed increased levels of HSP 27 and HSP90, showing that the above HSPs were ATP-independent chaperones.

The apoptosis procedure was altered with the initiation of intracellular endonucleases and proteases. The activation of several protease enzymes could improve the permeability of the mitochondrial membrane, including the ubiquitin-proteasome, calpain, and membrane-bound lysosomal enzymes, which further led to the release of cytochrome c [37]. Cathepsins, as the proteases localized in lysosomes, could respond to certain signals and activate caspases via the mitochondrial pathway with its release from lysosomes into cytoplasm. The study also showed that cathepsins were necessary for the release of pro-apoptosis factors from mitochondria to cytoplasm [14]. It was reported that cathepsin B and D participate in the regulation of cellular apoptosis [17,38]. This present research showed markedly increased activity of cathepsin B, L, and D in postmortem fish muscle, suggesting that the membrane of lysosome is ruptured and results in the increase in cathepsin activities in fish muscle. The increase in cathepsin (B and D) activities was also observed in postmortem bovine muscle [14]. The readily released lysosomal cathepsins from lysosomal membranes subsequently mediated the apoptosis procedure with the various stimuli during postmortem conditions. The study also showed that cathepsin D plays an important role in mediating apoptosis induced by cytokines tumor necrosis factor alpha (TNF-α) [38]. Previous research in grass carp muscle showed marked increased gene expression of TNF-α at 48 h [5], suggesting that the pro-inflammatory cytokines that mediated apoptosis may be enhanced by the release of cathepsin D from lysosome. Activation of Ca2+-dependent calpain may have an important role in apoptosis execution. Research showed that activation of calpains led to the degradation of nuclear substrates, cytoplasm, and membrane, causing the damage to cellular architecture and the apoptosis process. The mitochondrial structural changes and apoptosis were observed in fish muscle [12,33], which could also be partly induced by the enhanced activity of calpains at 24 h and 48 h. The increase in calpain activities was also observed in postmortem bovine muscle [14]. Serine proteinase inhibitor could prevent the activation of both caspase-3 and Fas-mediated apoptosis, suggesting that cytoplasmic serine proteinase participates in Fas-mediated apoptosis and caspases reactions. This study observed the increased activities of serine proteinase in postmortem grass carp muscle, implying that the potential role of serine proteinase was involved in the death receptor pathway in fish muscle. Combining the results, activation of protease is one of the factors that influences the activation of the apoptosis process in postmortem muscle. However, more studies are needed to elucidate regulation mechanisms of cathepsins, calpain, and serine proteinase involved in the apoptosis process.

## 5. Conclusions

**Figure 7:**
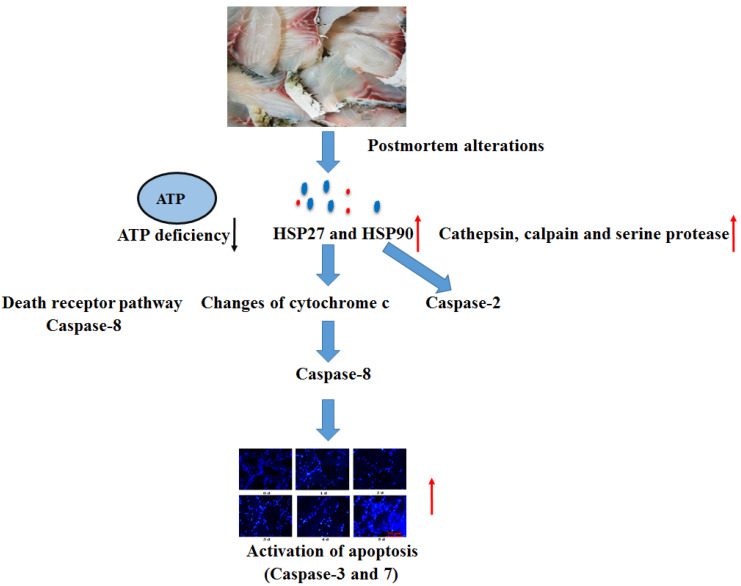
The schema showing the mechanism activation of apoptosis in fish muscle when stored at 4 °C for 120 h [14,26,38].

Activation of apoptosis and the inducing factors involved in the changes in flesh quality were systematically studied (Figure 7). The present study demonstrated that the activation of apoptosis occurred with the increase in storage time in postmortem grass carp muscle, evidenced by the increase in apoptotic nuclei. Positive apoptotic nuclei were positively correlated with drop loss and negatively correlated with shear force and water holding capacity, indicating that apoptosis was involved in the changes in flesh quality. The up-regulated transcriptional levels of caspase-2, 3, 7, 8, and 9 were also observed in different postmortem fish muscle, suggesting that the death receptor pathway and mitochondria apoptotic pathway were involved in the apoptosis process. The initiation of apoptosis was due to a decrease in intracellular ATP, the changes in cytochrome c redox state, as well as the increased protein levels of HSP27 and HSP90 and enhanced activity of endogenous proteases. The above results indicated that multiple apoptosis inducing factors and caspase transcriptional expressions played an active role in apoptosis alterations.

## Figures and Tables

**Table 1:** Real-time PCR primer sequences for studied genes.

Genes	Reverse Primer Sequence (5′→3′)	Forward Primer Sequence (5′→3′)	Temperature (°C)
β-actin	GGGCATAACCCTCGTAGAT	GGCTGTGCTGTCCCTGTA	61.4
caspase-2	ACGCCATTATCCATCTCCTCTC	CGCTGTTGTGTGTTTACTGTCTCA	60.3
caspase-9	GTGCTGGAGGACATGGGAAT	CTGTGGCGGAGGTGAGAA	59.0
caspase-8	TCCATCTGATGCCCATACAC	ATCTGGTTGAAATCCGTGAA	59.0
caspase-7	CCTTATCTGTGCCATTGCGT	GCCATTACAGGATTGTTTCACC	57.1
caspase-3	TCTGAGATGTTATGGCTGTC	GCTGTGCTTCATTTGTTTG	55.9

**Table 2:** Correlation coefficients (r) and the significance (*p*) of apoptosis and flesh quality indicators.

	Shear Force	Water Holding Capacity	Drop Loss
Positive apoptotic nuclei	−0.942 *	−0.973 *	0.982 *

* indicate *p* < 0.05.

**Table 3:** Correlation coefficients (r) and the significance (*p*) of apoptosis and related inducing factors.

	Cathepsin B	Cathepsin L	Cathepsin D	Calpain	Serine Proteinase
Positive apoptotic nuclei	−0.24	−0.06	0.07	−0.71	−0.28
	HSP 27	HSP 90	caspase-2	caspase-3	caspase-8
	0.95 *	0.89 *	0.96 *	0.48	−0.50
	Caspase-9	Oxidative cyt-c			
	−0.81	0.93 *			

* indicate *p* < 0.05.

## Data Availability

The data presented in this study are available on request from the corresponding author.
